# Fitness for Purpose in Online Communities: Community Complexity Framework for Diagnosis and Design of Socio-Technical Systems

**DOI:** 10.3389/fpsyg.2021.739415

**Published:** 2021-09-27

**Authors:** Agnieszka Rychwalska, Magdalena Roszczyńska-Kurasińska, Karolina Ziembowicz, Jeremy V. Pitt

**Affiliations:** ^1^ Robert Zajonc Institute for Social Studies, University of Warsaw, Warsaw, Poland; ^2^ Institute of Psychology, The Maria Grzegorzewska University, Warsaw, Poland; ^3^ Department of Electrical & Electronic Engineering, Imperial College London, London, United Kingdom

**Keywords:** collective action, socio-technical systems, social complexity, emergence, collective awareness, social self-organization, ICT

## Abstract

Recent discourse on Information and Communication Technologies’ (ICT) impact on societies has been dominated by negative side-effects of information exchange in huge online social systems. Yet, the size of ICT-based communities also provides an unprecedented opportunity for collective action, as exemplified through crowdfunding, crowdsourcing, or peer production. This paper aims to provide a framework for understanding what makes online collectives succeed or fail in achieving complex goals. The paper combines social and complexity sciences’ insights on structures, mechanics, and emergent phenomena in social systems to define a Community Complexity Framework for evaluating three crucial components of complexity: multi-level structuration, procedural self-organization, and common identity. The potential value of such a framework would be to shift the focus of efforts aimed at curing the malfunctions of online social systems away from the design of algorithms that can automatically solve such problems, and toward the development of technologies which enable online social systems to self-organize in a more productive and sustainable way.

## Introduction

Widespread adoption of Information and Communication Technologies (ICT) for social interaction and collaboration has brought about the promise for more ambitious and successful collective action (Benkler, 2016). Through new technologies, vast numbers of people can reach each other and coordinate with hardly any cost involved. The limits that existed on the size of possible organizations (Coase, 1937) are to a large extent removed (Shirky, 2008). Therefore, ICT-based collectives have the possibility of harnessing the inputs of huge numbers of contributors – orders of magnitude larger than is possible with offline socializing. Not only the volume of contributions grows but also the probability of tapping on rare skills, knowledge or potential.

It comes as little surprise then that many have hoped that new technologies will empower human collectives (Bimber et al., 2005; Booth, 2010; Zuckerman, 2014), liberate them from the limits of physical geography and nation-state based control (Cairncross, 1997; Goldsmith and Wu, 2006), and even transform their offline social contexts (Benkler and Nissenbaum, 2006).

High hopes for ICT based collective action have been fueled by the appearance of such successful projects as Wikipedia or Open Source Software (Linux, Firefox, Apache webserver, to name a few). Yet, many ICT mediated collective action initiatives – while seemingly attractive or important – never take off for good or never reach similar impact levels. For example, crowdsourcing of public policies – from participatory budgets, to strategical development plans – seem to never gather “crowds” (Liu, 2017). Carefully pre-designed online platforms often fail to gather a critical mass of contributors (Kraut et al., 2012). Most ICT-initiated activism, even if it is able to gather a critical mass in the short term, fails to reach its goals and to maintain any pressure on “real” life politics (Gladwell, 2010; Tufekci, 2014).

It is a challenge then to understand how ICT based collectives can reach their potential: take full advantage of their possible size and diversity to engage in, and accomplish complex tasks and functions. Such knowledge may become the basis for designing technological solutions that could help ICT to bring the much hoped for empowerment of collective action.

In this paper, we propose that complex tasks can be accomplished if the collective’s structure and mechanics produce coordination dynamics that matches the complexity of the task. We bring together social scientific concepts and complex systems perspective to define the Community Complexity Framework for assessing the collectives’ structuration and procedural self-organization together with system level, emergent properties such as identity and collective awareness. We argue that for online collectives, the affordances of technology together with their algorithmic back-end put constraints on what complexity of structures and coordination processes may occur.

The main contribution of our paper lies thus in providing a generic, systemic perspective on collective action mediated by social media. This perspective goes beyond the analysis of individual decisions to participate or volunteer (e.g., Bimber et al., 2005; Oreg and Nov, 2008), beyond designing and analyzing specific HCI tools for improved team work (e.g., Pitt et al., 2019), and beyond studying online governance models of collective action (e.g., Pitt and Ober, 2018). Instead, our specific contribution is to propose a framework for analysis of the mechanics of socio-technical systems: their structures and procedures, how these produce system dynamics in the form of specific collective behaviors, and how they are facilitated or constrained by the technology through which online collectives gather. Further, we exemplify the usefulness of the proposed framework by analyzing two cases of communities in which collective action of different complexity can be related to different levels of social self-organization that is constrained or promoted by specific technological choices. Finally, we propose to employ the Community Complexity Framework not only for analysis of existing communities but also as an initial step in specifying design principles for *supplying* sustainable online communities (cf. Ostrom, 1990). These tools and technologies would promote intentional design of socio-technical systems capable of self-organization, including adaptation to changing social environments, resistance to detrimental behaviors, and reflective self-awareness that can help with a continuous process of systemic improvement.

## Task Complexity

Arguably, people form groups and communities to be able to achieve more than they could achieve alone (Caporael, 2001; Brewer and Caporael, 2006). A long standing line of research on social facilitation shows that, in some conditions, groups outperform individuals (Allport, 1920; Hill, 1982; Bond and Titus, 1983). Some goals require a critical mass of others or a critical set of competences – therefore, collective action and collective intelligence may overcome obstacles that individuals cannot tackle (Bimber et al., 2005). A diverse set of goals can be achieved with collective action: from raising funds, to political activism, to such complex and challenging processes as changing law and societal norms (Hardin, 1982). Collective action online has also aimed at a diverse set of goals, from fund rising, to building knowledge bases, to inciting social movements (Zuckerman, 2014). Not surprisingly, the ambition of the goals is often reflected in the tasks undertaken by the collective action participants or community members.

We propose that the defining dimension here is complexity: as the intuitive perception of task difficulty grows, there are changes in what is required to be done. Individual tasks grow more difficult and more diverse and they are increasingly related to each other. Moreover, as the individual tasks diversify, more and more coordination is required to combine them into a meaningful whole. Rather than being simply aggregated – summed or averaged – they need to be arranged into an intricate patchwork. Thus, we may summarize this increasing inter-dependence between individual actions as increasing complexity of the collective function.

In principle it might be possible for a spontaneously, *ad hoc* organized social system – let us say, a flash mob – to perform a complex task. However, there is certain regularity in that more mature and long-standing communities often tackle more complex goals. For example, groups of activists tend to form NGOs after recurring actions (Staggenborg and Staggenborg, 1988; Alonso and Maciel, 2010), fundraisers grow effective management structures as they gather experience in repeated events (Herman and Renz, 1998) and businesses grow and develop more complex products after initial success (Hobday, 1998). In all these examples, experience enables achieving more complex goals but it is often accompanied with an evolution of the social systems – not only the goals change, but also the complexity of the group as well. In advanced online social systems – those that withstood the test of time (OSS, Wikipedia) – changes in management, task distribution, and technology have been accompanying the growth of the complexity of the artifact (O’Mahony and Ferraro, 2007; Butler et al., 2008). To be able to pinpoint what it is exactly that allows these more evolved collectives achieve complex tasks, we propose to analyze them as complex systems.

Complexity science is well-suited to provide generic concepts: it tries to find commonalities in mechanics between diverse systems (Miller and Page, 2009). This generic character of complexity concepts is very fitting to the analysis of online social systems whose functions and modes of operation can be extremely diverse: from gathering funds, through citizen science to writing code; from top down, proprietary platforms “ruled” by CEOs, to bottom up initiatives with distributed leadership. Complexity science can help find the commonalities and develop guidelines applicable to a variety of social institutions. Moreover, modeling approaches in complexity, specifically agent based modeling (ABM), focus on identifying the crucial properties of elements of the system and of the driving mechanics governing the systems dynamics (Gilbert, 2008). Such an approach can be a source of insights in the design of socio-technical systems.

Following this choice of theoretical grounds, we can propose that any social system, whether small or large, online or offline is composed of individuals that form a network of relations with one another and through them are able to influence the behavior of their alters. The composition and interactions within a social group are what determines whether the activity of the group will proverbially be more than the sum of its parts: “(…) complexity emerges as a result of the patterns of interaction between the elements” (Cilliers, 1998, p. 5). Structure, (often non-linear) interactions, and emergent dynamics are what define a complex system (Simon, 1962).

## Community Complexity Framework

### Structural Composition

When analyzing any organization or social group as a system, the first step is to identify its structural properties: the elements and the relations between them. Those structural properties are what enable interactions within the system and thus the dynamics of the system (Cilliers, 1998). By “identifying elements,” we do not mean identification of the precise individuals that comprise a system but rather its general composition.

In complexity science and agent based models of social systems elements are often thought of as average individuals who behave in the same way as all their peers. More advanced models recognize the heterogeneity of individuals, fitted for a particular application – for example, when modeling opinion changes in a society, elements can be divided into conformists and anti-conformists (Jarman et al., 2015). Differentiation of elements in a system is considered as conducive to more complex functions (Nowak et al., 2017). Element heterogeneity increases the complexity of the system and is adaptive in complex environments (Miller and Page, 2009). While such composition assumptions are more and more often drawn from experimental social studies, models by necessity are simplification of the true social diversity, as extensively studied in social sciences.

In social sciences, structures have been studied at many levels: from dyads (e.g., marriages or close relations), to small groups, to formal and informal organizations, to whole societies; and in many perspectives: from the psychological perspective underscoring individual differences in personality traits and predispositions, to informal group roles, to formal organizational positions, and to social classes in societies. Most importantly, many of those fields of studies explicitly investigate the effects of particular structural composition on the functioning of social systems. For example, compatibility or similarity of traits (i.e., element properties) within marriage leads to a quality relationship (Gonzaga et al., 2007). In groups and organizations, composition of roles is what determines how the system will function: “groups, organizations, and societies function by differentiating sets of tasks, each of which is assigned to or assumed by particular individuals” (Turner, 2001, p. 233).

In organizations these roles tend to be formalized in such a way as to rationally optimize function, wherein each element (individual) fulfils a role and can be replaced by any individual that can perform the same part (Waters and Waters, 2015). In management studies (Belbin, 2010) and psychological small group research (Benne and Sheats, 1948), formal roles are accompanied by less formalized behavioral patterns: functional roles (Benne and Sheats, 1948). Balance in the composition of such roles – i.e., leader, mediator, counselor, devil’s advocate, and so forth – is recognized as a factor of team effectiveness (Benne and Sheats, 1948; Prichard and Stanton, 1999; Belbin, 2010). Extant research thus shows that the diversification of formal roles and heterogeneity of individual properties are both typical for more complex systems as well as identified as factors impacting a social system’s operation.

### Connectivity Structure

Elements in a system are linked by relations that enable them to interact with each other and impact each other’s behavior and functioning: connections are what form a system from a loose assembly of individuals. The various setups of this interconnectivity have been extensively studied in complex systems with network analysis (Newman, 2010). Network studies have shown that diverse systems can have similar connectivity – for example, so called small world networks have been found in neural systems, energy transmission infrastructure, or actor co-play networks (Watts and Strogatz, 1998). Global properties of system’s connectivity, such as power law distribution of the number of connections elements have, can also be identified in many systems – social groups, the World Wide Web, cellular metabolic networks, and many others (Barabasi and Bonabeau, 2003). These global, ubiquitous properties of systems’ connectivity have been identified as some of the most important factors that impact the dynamics of the system.

In social sciences structures of relations between individuals, or between various roles or positions, have been studied most thoroughly in organization studies and sociology. The classic Weberian structure of organization is a hierarchy of roles that describes both the interactions of specific roles in the performance of particular, organization specific functions but also the decision making structure that optimizes effectiveness of the organization in achieving its goals (Waters and Waters, 2015). An important aspect of this hierarchy is that particular functions performed by individuals assuming roles need to be coordinated. The coordination costs relative to performance costs grow with the size of the organization, which imposes a limit on the size of such hierarchical structures: at some point, the increase in performance due to increase in size is invalidated by the increase of coordination costs (Coase, 1937). Other types of linkage or relations between particular tasks and their management have been proposed as improvement on such hierarchical, functional models, e.g., project- and process-based management (Turner and Keegan, 1999).

In bottom up systems – grass roots organizations and activism – there is a debate whether hierarchical or horizontal connectivity is better for performance. Some argue that for efficient functioning (i.e., attainment of goals) even bottom up structures need a hierarchical organization, as it speeds up coordination and decision making (Gladwell, 2010).

A more generalized view of structures is present in sociological theories, for example, structure is conceptualized as generalizable procedures applied in the enactment of social life (Giddens, 1984), the intangible principles (“schemas”) that push individuals to recursively reproduce patterns of social behavior (Sewell, 1992). In this view, social structures have a causal role in the dynamics of societies.

Connections and diversification of elements together form what we might term the structure of the system (Simon, 1977). It consists of the elements, their specific qualities, the relations among them. Structures can be more or less complex. The more diversity, the more types of relations, the more levels of grouping (e.g., clustering) among the elements, and the more complex is its structure (Weaver, 1948). Moreover, systems can change – or rather evolve – their structures in time. Very often a social system starts as a small group of equals but as it grows in size and in experience, it diversifies and evolves a more complex connectivity (Sawyer, 2005).

Drawing from the importance of such defined structure in both complexity and social science, we can define the first dimension of community complexity as *structuration*. It manifests in division of tasks (Benne and Sheats, 1948), diversification of contributors and relations between them, formation of task related user roles (Turner, 2001), and their interrelations and appearance of meso-level management (i.e., a hierarchy of roles). Increasing structuration allows developing a governance system with different privileges, responsibilities, and accountability schemes (Leskovec et al., 2010), for division of labor and coordination of work (Benkler, 2002), as well as for management of the community as a whole and its parts (Shaikh and Henfridsson, 2017).

In online social systems, diversification of elements and the types of connections between them are restricted by the technology. At one extreme there will be systems recognizing only one type of users, where contributions from one individual will be exactly the same and treated as equal as that of any other individual (e.g., when rating products). At the other extreme, we may have rich technology that allows diversification on many dimensions (tenure, reputation, groups for specific areas of activity or specific topics, followers and subscribers *vs.* followed and contributing, formal roles of admin, moderator, etc.).

While in offline social systems, relations can be both formal or abstract and can span time and space (e.g., friendship, trust, reputation, subjugation, domination, *etc.*), in online systems they are more concrete and their type is restricted by what is implemented into the technology (e.g., messages, friends lists, co-activity in certain areas of the platform, liking, attribution of karma points *etc.*). The more developed is the platform the more types of relations can be traced in a collective; at one extreme there will only be links that the individuals may not even be aware of (e.g., liking the same product by two shoppers), at the other – an open ended platform that allows the users themselves to define new types of relations, e.g., by designing new areas of activities.

### Rules and Interaction Mechanics

To define a complex system besides structure one needs to know the mechanisms by which elements influence each other along the existing connections (Miller and Page, 2009). In complexity science, influence is described by a function that operates on the inputs from other elements and which produces a change in the state of the given element (Gilbert, 2008). In the simplest form, it is the sum or average of the incoming (possibly weighted) signals, subjected to some threshold function. Depending on the modeled system, this function can be freely elaborated, to mimic anything from the impact of electric signals on synaptic neuronal connections, to conformism within a group, to the complex interplay of institutions regulating financial markets.

Such interaction rules often draw from the enormous body of theoretical and empirical research on social interactions in social sciences. In psychology, social influence is defined as any change in behavior or state of an individual that is due to real or imagined presence of others (Allport, 1954). This broad definition has led to investigation of such diverse phenomena as conformism, opinion leadership, intergroup conflicts, and persuasion techniques, among others (Asch, 1956; Cialdini, 1993; Tajfel and Turner, 2004). Mechanisms and sometimes precise functions that describe when and under what circumstances the change in behavior happens have been proposed (Latane, 1981).

In groups, informal rules (such as norms) and formal regulation (e.g., law, written guidelines, etc.) govern interaction between individuals and define accepted behavior. The formation of such rules – be they codified practices or normalized patterns of interaction – is an important factor in many areas of societal functioning (Ober, 2008). Errors in these mechanisms, such as an abuse of regulations, or formation of ineffective interactions, due to e.g., corruption, most often result in malfunction (Clausen et al., 2011). Mechanics of interactions within a system (i.e., the rules) that lead to effective collective action and sustainability have been identified for institutions governing common pool resources (Ostrom, 1990).

Codification of procedures that led to successful collective action helps the community accumulate knowledge and reduce erroneous decisions in the future. As such its role has been recognized in historical accounts of democracy development (Ober, 2008) and in theories of societal evolution (Cioffi-Revilla, 2005). Codified rules and informal norms thus become a resource that builds a social system’s collective knowledge.

In institutions, rules and procedures, together with the structures described earlier (i.e., roles and connectivity) form bureaucratic organization. Both insufficient determination of procedures and roles as well as overwhelming bureaucracy may lead to ineffective action (Jullien et al., 2015).

In online social systems, mechanisms that determine interaction between system elements are to a large extent restricted by the technology itself. In the simplest form, there can be algorithms that combine user contributions without the users’ knowledge (e.g., for profiling and marketing) or which are oblique to users (e.g., ratings from other users may be aggregated into a final reputation score of a contributor according to a rule designed by the platform proprietor). The affordances of platforms are also a natural limit on how a person can act toward others (e.g., by verbal communication, images, videos, etc.) and they also define the bandwidth of interaction (i.e., if the impact can be enacted on many users at the same time, on selected groups or on individuals). Formal policies and terms of service also serve to put boundaries on the behavior of users. In most advanced platforms, there is a variety of mechanisms that are present to not only limit but also guide the behavior of individuals (e.g., netiquette).

We thus define the next component of complexity: *procedural self-organization*. It describes the codification of common knowledge, its formulation into procedures, rules, regulations, and policies but also less formal guidelines, specifically those related to collective action. Increasing procedural self-organization allows the community to manage knowledge on collective functioning (Mitchell and Nicholas, 2006), to store it and share it (which helps easily socialize new members) but also to stabilize it and stop it from changing erratically in response to short term collective experiences.

Procedural self-organization in ICT based communities is one of the important safeguards against the negative side effects of the growing variety of contributors. Extraction of the know-how and of best practices of community operation provides benchmarks for individual contributors and subgroups. It also helps easily identify contributions aimed at destabilizing the community, vandalism or abuse (e.g., procedures on establishing relevance of content stop fake news from appearing on the Wikipedia; Keegan and Fiesler, 2017). For advanced communities, procedures are also indispensable for self-regulation, i.e., conflict resolution (Kittur et al., 2007) as well as sanctioning and penalizing misbehavior, which is crucial for the management of collective action (Ostrom, 1990).

### Dynamics and Emergence

Structure and rules governing the possible coupling between elements of a system determine the dynamical regimes of the whole system (Simon, 1962). That is, the possible states of the system as a whole and the trajectory the system takes to travel between these points are defined by its construction principles: structure and mechanics. In effect, we may think of structure and rules as the potential of the system that – once activated – produces the dynamics of the system. This distinction is important in that structures and rules may be considered fixed (or rather, changing on a slow time scale) while dynamics provides the fast changes of elements’ states and system’s properties.

To analyze a complex systems’ dynamics usually there is no need to trace the state changes of each element. Rather, we may pick some aggregate properties – order parameters – and analyze their trajectory (Miller and Page, 2009). In complexity science, models often pick one or more variables to represent the overall state of the system: e.g., the average of states of all elements might be interpreted as the average opinion of individuals in a social system or a local field potential of neurons in a neural cortex area (Gilbert, 2008). For social systems, other variables can be chosen and interpreted in various ways to represent, e.g. sentiment, public opinion, social unrest, quality of life, GDP, and many similar (Miller and Page, 2009). In many complex systems, we may recognize recurring patterns in the dynamics of the system and self-organization of the elements’ states into spatio-temporal configurations (Sawyer, 2005). Self-organization is a common, emergent property in complex systems (Gilbert, 2002).

Studying simple self-organization in social systems can lead to interesting applications, e.g., analyzing traffic congestion patterns, or clustering of opinions, but what is most interesting about the nature of emergence in social systems is the complex emergent phenomena, such as norms, culture, values, or ethics, and sometimes negative phenomena such as herd mentality (Johnson, 2002; Sawyer, 2005).

Both simple (nominal) emergence, such as self-organization, as well as the more complex forms of emergence, may affect the system’s performance. Emergent properties are the “whole” that proverbially exceeds the “sum of the parts.” Organizational culture can be a predictor of a company’s success (Shahzad et al., 2012), and group identity determines both the functioning of individuals as well as group processes, including protracted conflicts (Tajfel and Turner, 2004). Collective agency – the internalization of group strengths – can substitute for individual agency, when the latter is threatened (Stollberg et al., 2015). Thus, emergent, group level properties – group identity, norms, culture, etc. – determine how well a social system performs.

These emergent properties are especially important as in them we can see a circular causality between the system level and the individual: the impact of emergent properties on the behavior of elements, sometimes called immergence (Gilbert, 2002). In social systems, norms are a clear example of such a case – they evolve as acceptable conduct is being negotiated among individuals but once established they limit the possible behavior of members of the community or organization.

Circular causality in social systems is perhaps best captured by the seminal work of Giddens and his notion of duality of the social structure: on the one hand, social structure gives context for meaningful interpretation of behavior of individuals, social groups, and institutions at various scales but on the other hand the enactment of such interactions between structural elements builds or evolves the structure itself (Giddens, 1984).

This process can be related to its simpler version in nature’s stigmergy: a process in which elements of a social animal herd change the environment (context) of the herd’s functioning and thus recursively impact their own behavior (Dorigo et al., 2000). What is however unique to human social systems is that their constituents are self-aware and can reverse engineer the emergence of certain social properties. We may call this process collective self-awareness – the ability to become aware as a group of how the rules and mechanisms of interactions lead to desired or undesired properties of the whole community (e.g., a competitive organizational culture or systemic racism) and to redesign these mechanisms in a process of planned emergence (e.g., through changing of organizational procedures or laws which form the mechanics of the system; Rychwalska and Roszczynska-Kurasinska, 2017).

Noting the importance of emergent properties, we define the third component of community complexity: *common identity*. We define it as an emergent construct manifesting in the formulation of common goals, amassing norms, ethics, ideologies, and values, and in the most complex systems also in collective awareness.

When identity grows, awareness may appear: individuals first realize that they are a part of a community, and then realize the community has common goals to which the individuals contribute, then acknowledge the common identity and what it brings and finally understand the way in which the community self-organizes and self-governs – i.e., the community gains self-awareness (Pitt et al., 2013).

Collective awareness, and especially self-awareness, enables the community to critically observe its own functioning and to reflect on the long term goals and the stability of the group. This in turn allows conscious designing of corrections in the self-organization process. Such corrections are critically needed when the context of collective action changes – e.g., due to disruptive developments in technology, to changes in the social demand for community artifacts or to growth in size. Therefore, we hypothesize this last component of complexity to be a prime source of adaptability and long-term sustainability of the collective.

Quantification of common identity is a challenge, but an approximation can be made by measuring the number of contributors that in any way display or promote community related information when they present themselves to either other community members or outsiders. Complementary measures might be developed to assess the fraction of community members relating to community mission, values, or goals in communication with others. Finally, assessing the prevalence of various tools which the community uses to describe its own functioning (i.e., databases, visualizations, and statistics) can help diagnose whether the community has gained collective awareness.

## Diagnosing Complexity

In this section, we consider both the issues related to “measuring” complexity according to the framework of the previous section, as well as to applying such measurement in practice, for which we will need a systematic methodology to enable a comparative analysis.

### Measuring Complexity

The three dimensions of community complexity can be employed to assess how well a community is suited to achieve its goals or perform its functions. Our main premise here is that community complexity needs to be fitted to task or goal complexity. Different tasks require different complexity of communities (e.g., crowdsourcing a solution or deliberation on a political topic would require lower complexity than developing software). Too much complexity may be as detrimental to performing a function as not enough of it: advanced structures and rules require more engagement from contributors (Halfaker et al., 2012) and could potentially kill young communities.

We note that indicators measuring each of the dimensions need to focus on community activities and properties that do not directly serve the main community function (i.e., meta-activities). While communities can vary vastly in terms of particular tasks (from fund raising to writing software) as they mature they all need to develop roles, rules, norms, and similar. Thus, community complexity framework enables us to compare online social systems that perform vastly different tasks, including open social systems and proprietary platforms.

We stress that community complexity goes beyond assessing or measuring governance in online communities. The problem of governance has been extensively studied for online social systems, specifically for open systems, and a plethora of different types of governance have been identified: democratic, bureaucratic, ad-hocratic, fully centralized, and others (O’Mahony and Ferraro, 2007; Butler et al., 2008; Konieczny, 2010). In our approach, we would like to address a more generic question: to what extent are meta-activities related to structure, mechanics and dynamics (i.e., indicators of the community complexity dimensions) present in the social system. Thus, we abstract from the question of which type of governance is fitting for a particular community. Rather, based on the review of complexity science principles and social scientific results and theories, we assume that the more complex the task, the more meta-activities there should be. Governance is a part of community complexity – a large and important part – but it does not exhaust the concept. Identity and values, interaction norms, informal roles (both task related and supportive), diversity in individual traits, tacit and explicit knowledge formation, collective awareness, and other indicators of community complexity may play a crucial part in the effectiveness, sustainability, and adaptability of the community.

The functions a community serves should determine the complexity of meta-activities the community performs. What might not be so readily visible is that the complexity of actions performed is also determined by the underlying technology on which the community operates. Code is crucial in defining what is possible and what is unimaginable in online social contexts, just as physical space constraints – architecture, for example – define the possibilities in offline interaction (Lessig, 2000). In an ideal situation, the functions of a community should define what needs to be done and the technology should provide the means to do what needs to be done. Thus, there needs to be a fit between the function of a community and the affordances of the interface and engine of the platform on which it operates (Galegher et al., 2014). Simple functions require less functionalities and more rigid algorithmic framework that takes over many activities from the community members. Complex functions need more affordances as well as more flexible and open-ended algorithmic backbone that would allow contributors to act on their creativity and agency. Collectives requiring different levels of complexity will face different challenges in developing appropriate technological solutions to support the necessary meta-activities.

While well-fitted technology is definitely not a sufficient condition for a successful community, communities should be able to grow and mature with the help of good technology rather than in spite of its shortcomings, leading to more successful and sustainable communities. We propose that diagnosis of a community’s complexity should be followed by a careful analysis of the given technological environment and how it promotes appropriate complexity of the social system. For this diagnosis, and to support a comparative analysis, we need a methodology, as outlined next: this methodology will be applied to the two case studies of “Comparative Case Study: Kickstarter vs. Wikipedia.”

### Case Study: Methodology

The Community Complexity Framework provides a theoretical backbone for a multitude of methodologies to study the effectiveness of online collectives: from designing quantitative metrics to measure each dimension, to deep qualitative analysis of technological constraints on the dimensions, to scenario generation and prototype testing, and to large-scale computer simulations allowing virtual testing of the impact of particular functionalities on community complexity. While this paper focuses on presenting the framework as well as its justification in the findings of the complexity and social sciences, as an illustration of the usefulness of the framework in the next section we present results of a small-scale, qualitative case study. Our goal with the following analysis was to determine whether we can pinpoint which technological solutions promote or inhibit community complexity in mature (long-standing) communities with well-defined goals.

As the cases for the analysis we chose English language Wikipedia and Kickstarter: both are well known to wider public and thus do not need longer introductions that would go beyond the scope of our paper, and both are long-standing communities that can be considered successful in pursuing their goals. It is thus interesting to test whether the Community Complexity Framework can help diagnose differences in such well-developed communities and relate them to particular technology choices.

To answer this question, we qualitatively diagnosed the social self-organization of these communities as well as the functionalities of their respective platforms by analyzing their websites: for Wikipedia, focusing on the so called “project namespace” where community functioning is organized, as well as “user namespace,” where users self-present and send direct messages to each other; for Kickstarter, focusing on site policies and etiquette, as well as the project interface where backers and creators communicate. For Kickstarter, we have also qualitatively analyzed a sample of projects from the “Games” and “Design & Tech” categories (both create artifacts) selected from the top of the “Projects We Love” ranking to get a glimpse of the communication between backers and creators. Two researchers analyzed Wikipedia (AR and KZ) and two Kickstarter (AR and MR-K), one researcher checked and commented on the conclusions of the coders (JP).

The analysis focused on diagnostic measures of the community complexity dimensions, as presented in Community Complexity Framework: (a) for structuration: existence and prevalence of uptake of formal and informal roles, explicit and implicit relations between contributors, division of tasks, and contributor differentiation; (b) for procedural self-organization: existence of clearly defined procedures for community contribution, for coordination of activities, and for interaction between community members (including conflict resolution), as well as existence of detectible (e.g., in user communication) less formalized norms for behavior and interaction; (c) for common identity: expression of identification with the community by contributors, detectible expression of or reliance on community values, and evidence of community self-diagnosis.

## Comparative Case Study: Kickstarter Vs. Wikipedia

Kickstarter is one of the most prominent crowdfunding platforms. It boasts funding 207 thousand projects since 2009, recruiting 20 million backers and amassing 6 billion US dollars. When it first launched its main goal was to “(…) help you raise money for your ideas” but the motto evolved through “A new way to fund ideas & endeavors” and “Fund and follow creativity” up until its current goal of “Bringing creative projects to life.^1^” It thus strives to perform a complex task of supporting innovation – a generic goal that can be decomposed into many interdependent tasks (e.g., inventing an idea that answers a need, gathering necessary funds for the implementation, producing, distributing, and promoting the creation). The technological innovation behind Kickstarter is that of gathering a critical mass of micro investments through an online platform. The main functionalities include displaying, browsing, and searching through projects and deploying funds.

The English language Wikipedia is the largest, collaboratively created online encyclopedia. As of writing this paper, it has almost 6.2 million articles and over 40 million registered users. The goal of providing “Free access to the sum of all human knowledge^2^” is a complex one, comprising various tasks related to each other (amassing knowledge, writing articles, finding sources, categorization and building semantic relations between articles, formatting and copyediting, and many more). The technology behind this community is the open source MediaWiki platform that allows collaborative editing of documents, categorization, searching, and archiving.

Structuration in these two communities can be quantified by measuring the number of different roles and substructures in the social system. We can assess the structuration of Kickstarter community as a basic one. There are two dimensions on which the users are diversified: area of activity (i.e., projects) and user category. The first is provided by a hierarchical division of the whole platform into separate categories and then projects. The second is built by distinguishing the roles in the community: backers, creators, and the support team of the service provider. Relations and interdependencies between the user roles are possible only within projects; while some partake in many and it is possible to list all the projects a user contributed to, there is no direct way to bridge – through communication or otherwise – the separate parts of the platform. Thus, while in theory, the social network could be connected it is far more probable that it is divided into disjoined components. Moreover, there is very little overlap between the backers and creators groups, further deepening the divides. The main technological functionalities for structuration include hardwired user roles, communication through comments (for backers) and through updates (for creators).

Upon first glance, it might seem that the structuration levels of the Wikipedia community are not much higher. Just like many other platforms, the MediaWiki has inbuilt user roles that are common across many content management systems: a hierarchical user ladder starting from unregistered contributors (who have the right to participate) through registered ones up to administrators and bureaucrats. The roles are linked to specific privileges (capacities to perform certain actions like blocking other users from editing, checking IPs, or protecting certain content from edits).

Yet, this simple structure hard coded into the platform does not constitute the full structuration. Thanks to the open-ended character of the platform, the Wikipedians have designed a plethora of functions and task forces that are defined and described in the documents in the project namespace. Some of these structures are topic related, e.g., WikiProjects – groups of editors working on articles on specific themes. There are also function related groups, like counter vandalism unit or copyediting unit that by definition span all semantic categories. Moreover, roles are not only visible as divisions into sub-groups but also as individual specialization presented in the user namespace – e.g., some users focus on correcting failing links while others engage heavily in peer reviews of articles. The social structure is complex and allows for intricate coordination of individual activities. The main functionalities for structuration include platform defined user roles, and open-ended documents for definition of other roles as well as various means of communication (talk pages, direct messages, newsletters, mass-messaging, and forums).

Procedural self-organization may be approximated by assessing the number of separate rules or guidelines in operation in a community. As an example, only very basic procedural self-organization is present in the Kickstarter community in the form of Q & A, guides, policies, terms of service, and some links to tutorials on how to create projects – all supplied by the service provider. Other community members do not participate in codification of knowledge. The functionalities include a help section (including etiquette), FAQ for each project, and diversified privileges for contacting others dependent on user roles. The lack of affordances for knowledge creation and codification within the platform led to the formation of outside knowledge repositories to which users may refer in their communication on project comment pages, e.g., kickscammed.com or gofraudme.com, which help users spot fraudulent crowdfunding projects on Kickstarter.

In comparison, on Wikipedia, the open-ended character of the platform allowed the community to develop a complex set of rules that govern the behavior of individuals and the interactions between them. In the project namespace, together with the definition of structures, are the extracted procedures for effective operation of the community. They vary with respect to the area covered: how to edit and contribute, how to become an active member of the community, where to seek help (technical and social), how to gain a role (e.g., of an admin), or how to contribute in decision making. Technology for procedural self-organization includes open-ended documents for specification of rules and guidelines, privileges for specific user roles, and open communication for deliberation on rules.

On Kickstarter, the identity of the community members is fractured. While all visitors have probably seen the overall platform mission, the contributors do not seem to identify with it. Our qualitative analysis of the comments section in our selection of projects from the Games and Design & Tech categories showed that the identity of backers is that of shoppers placing “orders.” They do not feel a part of a community that brings creativity to life, they do not acknowledge the risks involved in funding innovations – in a large part they are customers ordering products with a delayed delivery estimate. On the other hand, creators treat the platform as a new form of venture capital – majority is businesses (including many start-ups). While some run many campaigns for consecutive products, scarcely any become backers of other projects.

Collective awareness on Kickstarter is only rudimentary. Contributors can become aware of others that funded the same project – if they check a user’s profile. There are certain technological affordances possibly aimed at boosting both identity and awareness – “projects we love” filter, spanning all categories; popular projects listing based on user visits and likes; ability to follow creators; and newsletter with team picks – but their impact is not visible in users’ communication. Diagnostics is limited to a listing of successful projects.

In contrast, common identity on the Wikipedia is very strong. Contributors to Wikipedia call themselves Wikipedians to highlight the distinct identity they possess as a collective. The common general goal and values are clearly stated in the Wikipedia purpose statement: “Free access to the sum of all human knowledge.” The common, mature identity is also visible in the prevalence of norms: from how and where to leave messages for one another, through understanding what are the acceptable opinion statements – in discussions and polls – up to developing a shared sense of humor, easily seen in some of the user contributed essays.

Not surprisingly, Wikipedians also have a very high level of collective awareness – the community itself designs its governance and analyses its own operation. From deciding on actions taken to promote Wikipedia and gathering money, to blackouts in response to major socio-political events – all the governance related procedures are subject to collective decision making. Apart from conscious design of its own operation, self-awareness on Wikipedia is also visible in the proliferation of diagnostic tools that enable users to observe how well the project functions, from visitor statistics to analytic tools for user promotion procedures. The technological functionalities include user tags (so called userboxes) displayed in the user namespace to show identification with various projects or user groups, open communication for discussing collective action, archiving system (i.e., database dumps storage), and a plethora of analytical tools and bots developed by users using MediaWiki API.

The communities on Kickstarter and Wikipedia have tackled their increasingly complex tasks in different ways. While both communities started small and both evolved their missions (from helping in gathering funding to support of creativity for Kickstarter and from an online encyclopedia edited by experts to the sum of human knowledge for Wikipedia), only Wikipedia has adjusted its structures and mechanics to fit the task. Kickstarter’s early implementation idea hardly changed since its inception: a front page composed of snapshots of selected projects with their statistics, a link to browse and discover projects, login functionality, micropayment solutions to gather funds, and commenting under projects. The graphical design and the implementation of the platform might have followed the evolution of web design, but the actual functionalities have not changed much. In effect, no matter the lofty mission statement, the actual collective action is nothing more than gathering funds.

On the other hand, observing the maturing of the Wikipedia community shows how the growth of community complexity can help tackle some of the problems brought about by task complexity as well as by the ICT enabled size of the community. The first step on this path was opening the project to contributions from non-experts. This led to demeaning of Wikipedia’s quality and possible role by the academia (Tumlin et al., 2007), but in spite of it, the popular interest started to grow rapidly. Since the original policy was that anyone can edit, there appeared many newcomers and many edits that were detrimental to the project. The hard working, engaged contributors were overwhelmed with a flood of bad content (Halfaker et al., 2012). In response, additional rules and policies were introduced. For example, unregistered users can no longer create new articles, there are speedy deletion procedures in cases of content falling far behind acceptable quality and there is a counter vandalism unit – users that constantly monitor suspicious activity. Moreover, technological affordances for quality control grew – e.g., in the form of bots that automatically revert edits algorithmically identified as malicious. An interaction space available to all contributors (forums) allows the community to diagnose problems linked to the complexity of the collective action and respond to them.

In sum, while both communities have withstood the test of time and maintained or even expanded their activities, the Community Complexity Framework allowed us to pinpoint differences in self-organization of the two collectives. Most importantly, there seems to be a mismatch between the ambitious and complex goal of Kickstarter (“bringing creative projects to life”) and the limited self-organization of the contributors: fractured communication, disjoint user groups, and lack of knowledge codification or of common identity. This low complexity of collective action is reflected (and possibly in part due to) limited platform functionalities, specifically, very few and restricted means of user communication. We might conclude that Kickstarter’s current motto is serving a role of an advertisement of the platform, rather than a true goal for the contributors involved. Social organization on the platform is better fitted to the original goal of “helping you raise money for your ideas,” which the community successfully fulfills: structuration into project oriented user groups, procedures (etiquette) on how to interact with backers and creators, both supported by appropriate functionalities on the platform, are well fitted to the task of amassing funds for particular ideas.

In contrast, self-organization of the Wikipedia community seems to be a good fit for its long term mission of amassing all of human knowledge, with high heterogeneity in both contributor roles and interaction channels, with a broad set of procedures and norms, and with a strong identity. This complexity of social organization is related to at least three specifics in technological foundations of the community: a varied set of interaction modes (from direct messages, to forums, and to communication threads related to content), a plethora of bots and other algorithmic tools answering specific community needs, and an open-ended character of the backbone of MediaWiki: collaborative editing of documents, whose content can span anything from encyclopedic articles, to community policies, to user profiles. At least some of these solutions might have been overwhelming for newly formed community (including Wikipedia in its inception phase) but seem well-fitted for a mature one: the growth of available tools and communication modes followed the growing needs of the complex self-organization of Wikipedians.

## Implications for Research and Practice

Analysis of the cases of Kickstarter and Wikipedia shows that application of the Community Complexity Framework may help pinpoint the weaknesses of collective organization (i.e., a mismatch between complexity components and the function the community strives to perform) and their relation to the technology on which the community operates. So far a lot of engineering effort is devoted to developing technological tools and interfaces to help individuals connect and form communities. We notice that there is less work put into the technological solutions that would enable performing specific functions after a network of connections between community members has been established, and to adapt community function to the possibly evolving goals.

Such solutions should be in part specific to the function of a particular community, and in part should be generic – applicable to any collective. This second set of functionalities relates to the meta-activities of a community: the actions required to coordinate individual tasks, to resolve conflicts, to set common goals and similar, and which so far seems to be underdeveloped. A possible reason for this lack of effort in the area of meta-activity functionalities is that there is not enough research and theoretical models that would describe what is actually needed in terms of coordination for communities. The relevant literature focuses on dyadic interaction or relatively small virtual teams and organizations (e.g., Warkentin et al., 1997; Walther, 2012; Lisiecka et al., 2016) but technology for large-scale communities is less researched.

Our paper fills this gap, first, by contributing the Community Complexity Framework which provides clear dimensions to measure community meta-activities, to assess their fitness for a particular function, and relates them to the available technological affordances. Second, we exemplify in a simple case study how social-self organization and the resultant system dynamics can be constrained or promoted by technological choices. Further research could analyze more cases of communities to provide benchmarks for structuration, procedural self-organization, and common identity for different complexity of community functions. Moreover, quantitative analyses of indicators of the three components could be developed that could further simplify the diagnosis of communities.

Finally, our contribution is in proposing that the three dimensions can be used as guidelines for developing design principles for online communities, in a vain similar to Ostrom’s design principles for common-pool resource management (Ostrom, 1990). For example, a database of available functionalities that properly serve the three components could provide a best-practices roadmap for analyzing and designing community operation. Such research contributions could inform the design of novel technological solutions – both in the interface as well as algorithmic back-end – to foster community complexity. In effect, we propose a novel avenue for both researchers and practitioners that would focus on conscious design and planned emergence of complex collective behaviors. In this we follow in the footsteps of the pioneers of cybernetics (e.g., Ashby, 1952; Beer, 1959, 1981), however, enhancing their ideas of engineering institutions with principles derived from social and complexity sciences, and specifically fitted to online collectives. Such an approach would focus on fostering productivity, resilience, and sustainability of collective action through social self-organization rather than through developing new AI and autonomous algorithmic tools aimed at alleviating the negative side-effects of previous generations of algorithms.

## Discussion

The fascination with ICT-enabled collective action has been fueled by the appearance of such successful collectives as Wikipedia or prime Open Source projects – Linux, Mozilla, or Apache Group. However, not all ICT-mediated collectives achieve similarly complex goals or produce elaborate artifacts. Many struggle and never gain the capacity for effective collective action. In some, negative collective phenomena appear (polarization of opinions, filter bubbles, and echo chambers, among others) that further reduce the capability for effective collective action.

For collective input to gain novel qualities when amassed, it needs to come not from disconnected individuals but from a social system full of interactions between its constituents – a complex system (Sawyer, 2005). By analyzing collective action from the perspective of complex systems and combining it with the concepts developed and measured within social sciences, we were able to propose general dimensions for diagnosis of online collectives.

First, the complexity perspective let us separate the structure of a social system from its dynamics and to assign specific indices of each. While the structure and mechanics determine the possible dynamics, only concurrent analysis of both may lead to diagnosis of the potential of the system and the way this potential is enacted. Combining complexity and social science perspectives allows more formal and at the same time richer understanding and diagnosis of what makes collectives succeed. The second important advantage of such an approach is that it provides insights as to how to steer collectives toward more complex dynamics. While the flagship property of complex systems is that they exhibit complexity even when their mechanics is fairly simple, the reverse – negative self-organization – can also happen. For example, epileptic seizures in brain function dramatically reduce the richness of this extremely complex system. Similarly, in social systems interactions may lead to a decrease in plurality and complexity: herding, rich-get-richer phenomena, and more recently – due to the specifics of the new media for social interactions – filter bubbles (Pariser, 2011) and echo chambers (Rychwalska and Roszczynska-Kursinska, 2018). In these cases, the same system switches between regimes of dynamics (i.e., displays bi- or multi-stability) that differ vastly in complexity.

In complex systems, the problem of inducing a switch toward a more desirable regime is a problem of controllability (Daniels et al., 2017) – e.g., targeting a set of nodes from the outside to make them “lead” the change (Gao et al., 2014). Yet, arguably, developing an online social system in which such a vulnerability to outside control is present might be counter-productive. Fake news planting and propagation on current social media resemble exactly such a manipulation. Therefore, implementing algorithmic solutions to solve online social systems problematic dynamics might instead lead to a new set of problems. Social science brings an alternative, albeit challenging, solution: to construct the social complex systems in such a way that positive emergent processes are possible. If the social group can become aware of its own mechanics and the (sometimes undesirable) emergent properties, it might consciously redesign its own structure and mechanics. In effect, control lies within the system itself.

The final contribution of this paper is thus related to the possibilities of designing online social systems displaying such emergent properties. When describing the differences between offline and online communities we have stressed that for online collectives, the structure and rules of the system are often constrained by technology and the emergent properties can be traced in the digital records of community activity. On the one hand, this allows diagnosis of each of these components, on the other – conscious design and planning for the emergent qualities. Effectively, designing online collectives resembles creating an Agent Based Model *in vitro*. So far this has been hardly appreciated by platform designers, engineers, and service providers, who may lack the knowledge of either complex or social systems. In effect, the emergent properties of online collectives often evolve erratically. While the behavior of users – including unexpected usage – may impact the further development of a platform, this process is responsive in nature and does not allow for prediction and long term goal-oriented design. The exemplar comparison between two online collectives (Kickstarter and Wikipedia) pinpoints how limited technological solutions might impair the complexity of collective action – e.g., limited communication, stiff divisions, no means to store community experience, lack of possibility for diversification of contributors, and so forth. Kickstarter’s motto suggests that there is potential to increase the scope of the community task from fundraising to promoting creativity, but for such an increase in complexity of collective action, fitting technology should be developed.

We advocate for a conscious, goal-oriented and scientifically-informed design of technological solutions for online social systems. This is complementary to proposals for encoding deep social knowledge in the customization of social media platforms (Pitt et al., 2021). The synergy of these approaches might result in more innovative and generative products and services developed in collaborative way – indeed using the same tools and platforms – and may also mitigate or even better prevent some of the daunting, negative social dynamics visible in current social media.

## Data Availability Statement

The original contributions presented in the study are included in the article/supplementary material; further inquiries can be directed to the corresponding author.

## Author Contributions

AR: conceptualization, literature review, investigation, and original draft writing. MR-K, KZ, and JP: investigation, validation, and original draft writing. All authors contributed to the article and approved the submitted version.

## Funding

This work was supported by the Polish National Science Center under Grant 2017/01/X/HS6/00833.

## Conflict of Interest

The authors declare that the research was conducted in the absence of any commercial or financial relationships that could be construed as a potential conflict of interest.

## Publisher’s Note

All claims expressed in this article are solely those of the authors and do not necessarily represent those of their affiliated organizations, or those of the publisher, the editors and the reviewers. Any product that may be evaluated in this article, or claim that may be made by its manufacturer, is not guaranteed or endorsed by the publisher.

## Footnotes

^1^All Kickstarter mottos retrieved from web.archive.org.

^2^Wikipedia motto retrieved from https://en.wikipedia.org/wiki/Wikipedia:Purpose.

## References

[ref1] Allport F. H. (1920). The influence of the group upon association and thought. J. Exp. Psychol. 3, 159–182. doi: 10.1037/h0067891

[ref2] Allport G. W. (1954). The Nature of Prejudice. Oxford, England: Addison-Wesley.

[ref3] Alonso A. Maciel D. (2010). From protest to professionalization: Brazilian environmental activism after Rio-92. J. Environ. Dev. 19, 300–317. doi: 10.1177/1070496510378101

[ref4] Asch S. E. (1956). Studies of independence and conformity: I. A minority of one against a unanimous majority. Psychol. Monogr. Gen. Appl. 70, 1–70. doi: 10.1037/h0093718

[ref5] Ashby W. R. (1952). Design for a Brain. London: Chapman & Hall.

[ref6] Barabasi A. L. Bonabeau E. (2003). Scale-free networks. Sci. Am. 288, 60–69. doi: 10.1038/scientificamerican0503-60, PMID: 12701331

[ref7] Beer S. (1959). Cybernetics and Management. London: The English Universities Press.

[ref8] Beer S. (1981). Brain of the Firm. 2nd Edn. Chichester: Wiley.

[ref9] Belbin R. M. (2010). Management Teams. 3rd Edn. London: Routledge.

[ref10] Benkler Y. (2002). Coase’s penguin, or, Linux and “The nature of the firm.” Yale Law J. 112, 369–446. doi: 10.2307/1562247

[ref11] Benkler Y. (2016). Peer production, the commons, and the future of the firm. Strateg. Organ. 15, 264–274. doi: 10.1177/1476127016652606

[ref12] Benkler Y. Nissenbaum H. (2006). Commons-based peer production and virtue. J. Polit. Philos. 14, 394–419. doi: 10.1111/j.1467-9760.2006.00235.x

[ref13] Benne K. D. Sheats P. (1948). Functional roles of group members. J. Soc. Issues 4, 41–49. doi: 10.1111/j.1540-4560.1948.tb01783.x

[ref14] Bimber B. Flanagin A. J. Stohl C. (2005). Reconceptualizing collective action in the contemporary media environment. Commun. Theory 15, 365–388. doi: 10.1111/j.1468-2885.2005.tb00340.x

[ref15] Bond C. F. Titus L. J. (1983). Social facilitation: a meta-analysis of 241 studies. Psychol. Bull. 94, 265–292. doi: 10.1037/0033-2909.94.2.265, PMID: 6356198

[ref16] Booth D. R. (2010). Peer Participation and Software: What Mozilla Has to Teach Government. Cambridge, MA: MIT Press.

[ref17] Brewer M. B. Caporael L. R. (2006). “An evolutionary perspective on social identity: revisiting groups,” in Evolution and Social Psychology. eds. Schaller M. Simpson J. A. Kenrick D. T. (Madison, CT, US: Psychosocial Press), 143–161.

[ref18] Butler B. Joyce E. Pike J. (2008). “Don’T look now, but We’ve Created a Bureaucracy: The Nature and Roles of Policies and Rules in Wikipedia.” in *Proceedings of the SIGCHI Conference on Human Factors in Computing Systems CHI ‘08*; April 5-10, 2008; New York, NY, USA: ACM, 1101–1110.

[ref19] Cairncross F. (1997). The Death of Distance: How the Communications Revolution Will Change our Lives. Boston: Harvard Business School Press.

[ref20] Caporael L. R. (2001). “Parts and wholes: The evolutionary importance of groups,” in Individual Self, Relational Self, Collective Self. eds. Sedikides C. Brewer M. B. (New York, NY, US: Psychology Press), 241–258.

[ref21] Cialdini R. (1993). Influence: The Psychology of Persuasion. New York: Morrow.

[ref22] Cilliers P. (1998). Complexity and Postmodernism: Understanding Complex Systems. London: Routledge.

[ref23] Cioffi-Revilla C. (2005). A canonical theory of origins and development of social complexity. J. Math. Sociol. 29, 133–153. doi: 10.1080/00222500590920860

[ref24] Clausen B. Kraay A. Nyiri Z. (2011). Corruption and confidence in public institutions: evidence from a global survey. World Bank Econ. Rev. 25, 212–249. doi: 10.1093/wber/lhr018

[ref25] Coase R. H. (1937). The nature of the firm. Economica 4, 386–405. doi: 10.1111/j.1468-0335.1937.tb00002.x

[ref26] Daniels B. C. Krakauer D. C. Flack J. C. (2017). Control of finite critical behaviour in a small-scale social system. Nat. Commun. 8:14301. doi: 10.1038/ncomms14301, PMID: 28186194 PMC5309824

[ref27] Dorigo M. Bonabeau E. Theraulaz G. (2000). Ant algorithms and stigmergy. Futur. Gener. Comput. Syst. 16, 851–871. doi: 10.1016/S0167-739X(00)00042-X

[ref28] Galegher J. Kraut R. E. Egido C. (2014). Intellectual Teamwork: Social and Technological Foundations of Cooperative Work. New York: Psychology Press.

[ref29] Gao J. Liu Y.-Y. D’Souza R. M. Barabási A.-L. (2014). Target control of complex networks. Nat. Commun. 5:5415. doi: 10.1038/ncomms6415, PMID: 25388503 PMC4243219

[ref30] Giddens A. (1984). The Constitution of Society: Outline of the Theory of Structuration. Berkeley: University of California.

[ref31] Gilbert N. (2002). “Varieties of emergence.” in Agent 2002 Conference: Social agents: ecology, exchange, and evolution; October 11-12, 2002; Chicago, 11–12.

[ref32] Gilbert N. (2008). Agent-Based Models. London: SAGE.

[ref33] Gladwell M. (2010). Small change. The New Yorker 4, 42–49.

[ref34] Goldsmith J. Wu T. (2006). Who Controls the Internet?: Illusions of a Borderless World. New York: Oxford University Press.

[ref35] Gonzaga G. C. Campos B. Bradbury T. (2007). Similarity, convergence, and relationship satisfaction in dating and married couples. J. Pers. Soc. Psychol. 93, 34–48. doi: 10.1037/0022-3514.93.1.34, PMID: 17605587

[ref36] Halfaker A. Geiger R. S. Morgan J. T. Riedl J. (2012). The rise and decline of an open collaboration system: how wikipedia’s reaction to popularity is causing its decline. Am. Behav. Sci. 57, 664–688. doi: 10.1177/0002764212469365

[ref37] Hardin R. (1982). Collective Action. New York: Resources for the Future.

[ref38] Herman R. D. Renz D. O. (1998). Nonprofit organizational effectiveness: contrasts between especially effective and less effective organizations. Nonprofit Manag. Leadersh. 9, 23–38. doi: 10.1002/nml.9102

[ref39] Hill G. W. (1982). Group versus individual performance: are N + 1 heads better than one? Psychol. Bull. 91, 517–539. doi: 10.1037/0033-2909.91.3.517

[ref40] Hobday M. (1998). Product complexity, innovation and industrial organisation. Res. Policy 26, 689–710. doi: 10.1016/S0048-7333(97)00044-9

[ref41] Jarman M. Nowak A. Borkowski W. Serfass D. Wong A. Vallacher R. (2015). The critical few: anticonformists at the crossroads of minority opinion survival and collapse. J. Artif. Soc. Soc. Simul. 18:6. doi: 10.18564/jasss.2663

[ref42] Johnson S. (2002). Emergence: The Connected Lives of Ants, Brains, Cities, and Software. New York: Simon and Schuster.

[ref43] Jullien N. Crowston K. Ortega F. (2015). “The rise and fall of an online project: is bureaucracy killing efficiency in open knowledge production?” in *Proceedings of the 11th International Symposium on Open Collaboration OpenSym ‘15*; August 19-21, 2015; New York, NY, USA: Association for Computing Machinery, 1–10.

[ref44] Keegan B. Fiesler C. (2017). The evolution and consequences of peer producing Wikipedia’s rules. Socarxiv [Preprint]. doi: 10.31235/osf.io/28sgr

[ref45] Kittur A. Suh B. Pendleton B. A. Chi E. H. (2007). “He Says, She Says: Conflict and Coordination in Wikipedia.” in Proceedings of the SIGCHI Conference on Human Factors in Computing Systems CHI ‘07; 28 April 2007 - 3 May 2007; New York, NY, USA: ACM, 453–462.

[ref46] Konieczny P. (2010). Adhocratic governance in the internet age: a case of wikipedia. J. Inform. Tech. Polit. 7, 263–283. doi: 10.1080/19331681.2010.489408

[ref47] Kraut R. E. Resnick P. Kiesler S. Burke M. Chen Y. Kittur N.. (2012). Building Successful Online Communities: Evidence-Based Social Design. Cambridge, MA: MIT Press.

[ref48] Latane B. (1981). The psychology of social impact. Am. Psychol. 36, 343–356. doi: 10.1037/0003-066X.36.4.343

[ref49] Leskovec J. Huttenlocher D. P. Kleinberg J. M. (2010). Governance in social media: A case study of the wikipedia promotion process. Available at: http://www.aaai.org/ocs/index.php/ICWSM/ICWSM10/paper/viewFile/1485/1841/ (Accessed August 01, 2016).

[ref50] Lessig L. (2000). Code and Other Laws of Cyberspace. New York: Basic Books.

[ref51] Lisiecka K. Rychwalska A. Samson K. Łucznik K. Ziembowicz M. Szóstek A.. (2016). Medium moderates the message. How users adjust their communication trajectories to different media in collaborative task solving. PLoS One 11:e0157827. doi: 10.1371/journal.pone.0157827, PMID: 27337037 PMC4919010

[ref52] Liu H. K. (2017). “Crowdsourcing Design: A Synthesis of Literatures.” in *Hawaii International Conference on System Sciences*; January 3-7, 2017.

[ref53] Miller J. H. Page S. E. (2009). Complex Adaptive Systems: An Introduction to Computational Models of Social Life. Princeton: Princeton University Press.

[ref54] Mitchell R. Nicholas S. (2006). Knowledge creation in groups: the value of cognitive diversity, transactive memory and open-mindedness norms. Electron. J. Knowl. Manag. 4, 67–74.

[ref55] Newman M. (2010). Networks: An Introduction. Oxford: Oxford University Press.

[ref56] Nowak A. Vallacher R. R. Zochowski M. Rychwalska A. (2017). Functional synchronization: The emergence of coordinated activity in human systems. Front. Psychol. 8:945. doi: 10.3389/fpsyg.2017.00945, PMID: 28659842 PMC5468424

[ref58] Ober J. (2008). Democracy and Knowledge: Innovation and Learning in Classical Athens. Princeton: Princeton University Press.

[ref57] O’Mahony S. Ferraro F. (2007). The emergence of governance in an open source community. Acad. Manag. J. 50, 1079–1106. doi: 10.5465/amj.2007.27169153

[ref59] Oreg S. Nov O. (2008). Exploring motivations for contributing to open source initiatives: The roles of contribution context and personal values. Comput. Hum. Behav. 24, 2055–2073. doi: 10.1016/j.chb.2007.09.007

[ref60] Ostrom E. (1990). Governing the Commons: The Evolution of Institutions for Collective Action. Cambridge, UK: Cambridge University Press.

[ref61] Pariser E. (2011). The Filter Bubble: What the Internet Is Hiding from you. UK: Penguin.

[ref62] Pitt J. Bourazeri A. Nowak A. Roszczynska-Kurasinska M. Rychwalska A. Santiago I. R.. (2013). Transforming big data into collective awareness. Computer 46, 40–45. doi: 10.1109/MC.2013.153

[ref63] Pitt J. Ober J. (2018). “Democracy by design: basic democracy and the self-organisation of collective governance.” in 2018 IEEE 12th International Conference on Self-Adaptive and Self-Organizing Systems (SASO); September 3-7, 2018, 20–29.

[ref64] Pitt J. Rychwalska A. Roszczynska-Kurasinska M. Nowak A. (2019). Democratizing platforms for social coordination. IEEE Technol. Soc. Mag. 38, 43–50. doi: 10.1109/MTS.2019.2894459

[ref65] Pitt S. van Meelis Lacey M. Scaife E. Pitt J. (2021). No App is an island: collective action and sustainable development goal-sensitive design. Int. J. Interact. Multimed. Artif. Intell. 6, 24–33. doi: 10.9781/ijimai.2021.02.005

[ref66] Prichard J. S. Stanton N. A. (1999). Testing Belbin’s team role theory of effective groups. J. Manag. Dev. 18, 652–665. doi: 10.1108/02621719910371164

[ref67] Rychwalska A. Roszczynska-Kurasinska M. (2017). Value sensitive design for peer production systems: mediating social interactions. IEEE Technol. Soc. Mag. 36, 48–55. doi: 10.1109/MTS.2017.2728737

[ref68] Rychwalska A. Roszczynska-Kursinska M. (2018). “Polarization on social media: when group dynamics leads to societal divides.” in *Hawaii International Conference on System Sciences*; January 2-6, 2018.

[ref69] Sawyer R. K. (2005). Social Emergence: Societies As Complex Systems. New York: Cambridge University Press.

[ref70] Sewell W. H. (1992). A theory of structure: duality, agency, and transformation. Am. J. Sociol. 98, 1–29. doi: 10.1086/229967

[ref71] Shahzad F. Luqman R. A. Khan A. R. Shabbir L. (2012). Impact of organizational culture on organizational performance: An overview. IJCRB 3, 975–985.

[ref72] Shaikh M. Henfridsson O. (2017). Governing open source software through coordination processes. Inf. Organ. 27, 116–135. doi: 10.1016/j.infoandorg.2017.04.001

[ref73] Shirky C. (2008). Here Comes Everybody: The Power of Organizing Without Organizations. New York: Penguin.

[ref74] Simon H. A. (1962). The architecture of complexity. Proc. Am. Philos. Soc. 106, 467–482.

[ref75] Simon H. A. (1977). “The Organization of Complex Systems,” in Models of Discovery Boston Studies in the Philosophy of Science. eds. Cohen R. S. Wartofsky M. W. (Netherlands: Springer), 245–261.

[ref76] Staggenborg A. S. Staggenborg S. (1988). The consequences of professionalization and formalization in the prochoice movement. Am. Sociol. Rev. 53, 585–606. doi: 10.2307/2095851

[ref77] Stollberg J. Fritsche I. Bäcker A. (2015). Striving for group agency: threat to personal control increases the attractiveness of agentic groups. Front. Psychol. 6:649. doi: 10.3389/fpsyg.2015.00649, PMID: 26074832 PMC4444748

[ref78] Tajfel H. Turner J. C. (2004). The Social Identity Theory of Intergroup Behavior. New York, NY, US: Psychology Press.

[ref79] Tufekci Z. (2014). The medium and the movement: digital tools, social movement politics, and the end of the free rider problem. Policy Internet 6, 202–208. doi: 10.1002/1944-2866.POI362

[ref80] Tumlin M. Harris S. R. Buchanan H. Schmidt K. Johnson K. (2007). Collectivism vs. individualism in a wiki world: librarians respond to Jaron Lanier’s essay “digital Maoism: The hazards of the new online collectivism.” Ser. Rev. 33, 45–53. doi: 10.1016/j.serrev.2006.11.002

[ref81] Turner R. H. (2001). “Role theory,” in Handbook of Sociological Theory Handbooks of Sociology and Social Research. ed. Turner J. H. (Boston, MA: Springer), 233–254.

[ref82] Turner J. R. Keegan A. (1999). The versatile project-based organization: governance and operational control. Eur. Manag. J. 17, 296–309. doi: 10.1016/S0263-2373(99)00009-2

[ref83] Walther J. B. (2012). Interaction through technological lenses computer-mediated communication and language. J. Lang. Soc. Psychol. 31, 397–414. doi: 10.1177/0261927X12446610

[ref84] Warkentin M. E. Sayeed L. Hightower R. (1997). Virtual teams versus face-to-face teams: an exploratory study of a web-based conference system. Decis. Sci. 28, 975–996. doi: 10.1111/j.1540-5915.1997.tb01338.x

[ref85] Waters T. Waters D. (2015). Weber’s Rationalism and Modern Society: New Translations on Politics, Bureaucracy, and Social Stratification. New York: Springer.

[ref86] Watts D. J. Strogatz S. H. (1998). Collective dynamics of small-world networks. Nature 393, 440–442. doi: 10.1038/30918, PMID: 9623998

[ref87] Weaver W. (1948). Science and complexity. Am. Sci. 36, 536–544. PMID: 18882675

[ref88] Zuckerman E. (2014). New media, new civics? Policy Internet 6, 151–168. doi: 10.1002/1944-2866.POI360

